# Burden of leprosy in Malawi: community camp-based cross-sectional study

**DOI:** 10.1186/1472-698X-12-12

**Published:** 2012-08-06

**Authors:** Kelias P Msyamboza, Leonard R Mawaya, Hudson W Kubwalo, David Ng’oma, Moses Liabunya, Sunganani Manjolo, Pilirani P Msiska, Wilson W Somba

**Affiliations:** 1 World Health Organisation, Malawi Country Office, Lilongwe, Malawi; 2 Ministry of Health, Lilongwe, Malawi; 3 World Health Organisation, Malawi Country Office, ADL House, 2nd Floor, City Centre, P.O. Box 30390, Lilongwe, 3, Malawi

**Keywords:** Leprosy, Skin diseases, Sub-Saharan Africa, Malawi

## Abstract

**Background:**

Although leprosy was eliminated globally in 2000, the disease continues to be the significant cause of peripheral neuropathy, disability and disfigurement in some developing countries. However, recent population-based prevalence data are lacking to inform evidence-based renewed commitment for the final push for leprosy elimination at national and sub-national levels.

**Methods:**

Community camp-based cross-sectional descriptive study was conducted in four selected districts. World Health Organisation guidelines and tools for leprosy elimination monitoring were used to evaluate the Malawi National Leprosy Programme.

**Results:**

A total of 6,338 people (60% females, 35% children aged less than 15 years) were examined for leprosy and other skin diseases. Prevalence of skin diseases was 18%, the commonest being fungal (9%), eczema/dermatitis (3%) and leprosy (1%). Of the fungal skin conditions, pityriasis versicolor and Tinea capatis were the commonest (22% and 21% respectively) then Tinea corporis (9%), Tinea cruris (6%) and Tinea pedis (2%). A total of 66 leprosy cases were detected out of 6,338 people screened giving a prevalence of 104.1 per 10,000 population (range by district 67.1 to 194.1). Of the leprosy cases, 37 were new, 6 were defaulters and 23 were on treatment, 30 were females and 9 were children aged less than 15 years old. Of the 37 new leprosy cases, 9 (24.3%) were children, 25 (67.6%) had 1–5 leprosy lesions and 8 (21.6%) had grade 2 disability. The most frequent location of leprosy lesions was the head and neck (24.1%), arms (24.1%), chest (17.2%), legs (13.8%), back (13.8%) and abdomen (7.0%). Between 2006 and 2011, trends of leprosy prevalence and detection increased, prevalence/detection ratios were over 1 and cure rates by cohort analysis of 2009 multibacillary and 2010 paucibacillary cases were 33% and 63% respectively far below the expected 80% although the national prevalence remained at less than 1 case per 10,000 population.

**Conclusion:**

Leprosy was still an important public health problem in Malawi. Improving knowledge and skills of health workers, registration and recording of data, contact tracing, decentralisation and integration of treatment to health centres and introduction of leprosy awareness days and community-based surveillance could help to improve early detection, treatment, case holding and prevention of disabilities.

## Background

Leprosy also known as Hansen disease named after a Norwegian physician Gerhard Henrik Armauer Hansen who identified the causative organism in 1873, is a skin and nerve infection caused by *Mycobacterium leprae.* Since biblical times, the disease continues to be an important cause of peripheral neuropathy, disability and disfigurement [1]. Worldwide, 2 million people are estimated to be disabled by leprosy. In 2010, 228 474 new cases were detected and the worldwide registered prevalence was 192 246 cases [2,3]. Word Health Organisation (WHO) targeted leprosy as one of the diseases to be eliminated from the world as a public health problem by reducing the prevalence to less than 1 case per 10,000 population based on the use of multi-drug therapy (MDT). Despite the success of MDT, endemic pools still exist in some countries that attained the national elimination threshold [3-5].

Malawi attained the WHO leprosy elimination status in 1994. Nationally, it still maintains this status where in 2010 the country registered a total of 632 leprosy cases out of 14 million people, representing 0.5 cases per 10,000 population. However, by district, at least 6 districts were off target. These were; Nkhotakota (2.9), Ntchisi (1.2), Mchinji (1.1), Nsanje (1.1), Balaka (1.0) and Salima (1.0). Of 632 leprosy cases registered or on treatment in 2010, 88% had more than 5 skin lesions (multibacillary) indicating that patients presented late to health facilities, 57% were males and 15 (2.4%) were children under the age of 15 years. The presense of leprosy among children is an indicator of on-going disease transmission in the community. These leprosy cases were registered through passive surveillance whereby cases were self-reporting to health facilities with minimal or no promotion of community awareness by health workers. The actual prevalence of leprosy at community level and number of districts that were off elimination target were therefore likely to be higher than what was being reported by national programme. In addition, health system challenges which the National Leprosy and Skin Control Programme was facing (inadquate financial resources, trained health workers, transport to follow up patients, supportive supervision) were likely to contribute to under-reporting and under-diagnosis of leprosy cases [6]. Leprosy is endemic in all the 28 districts in the country.

Skin diseases are common in developing countries with community-based prevalence ranging from 20% to 80% [7-10]. However, recent population-based data on the prevalence and challenges of leprosy and other skin diseases are scarce in Malawi and sub-Saharan Africa in general. The WHO Goodwill Ambassador for Leprosy Elimination Mr Yohei Sasakawa visited Malawi in July 2011 to raise political and community awareness and commitment on leprosy. Among other issues, the Goodwill Ambassador recommended the evaluation of the national leprosy programme to document successes, challenges and proposed way forward. This study was therefore conducted to support Ministry of Health to determine the population-based prevalence and challenges of leprosy and other skin diseases as part of the evaluation of the national leprosy programme.

## Methods

### Ethics statement

Ethical approval was granted by the Malawi National Health Sciences Research and Ethics Committee. Written informed consent was obtained before participants were enrolled in the study. For children aged 5–14 years, written informed consent was obtained from their parents or guardians.

### Study design

This was a descriptive cross-sectional community camp-based study. It was conducted in two parts. Part one was leprosy active surveillance and promotion of community awareness conducted in four districts, two high and two low leprosy endemic districts. The main aim of part one of the study was to determine the population-based prevalence of leprosy. Part two was the the evaluation of the National Leprosy Elimination Programme. The main aim of part two was to assess burden and trends of leprosy and its challenges at national level and in selected districts. Both part one and two of the study were conducted in the same districts.

### Part one of the study: Leprosy active surveillance and promotion of community awareness

#### Selection of study sites and Sample size

In the selected districts of Salima, Machinga, Mangochi and Nkhotakota; 5 enumeration areas (EAs) were randomly selected in each district using probability proportional to size (PPS) sampling method. The minimum sample size was calculated using the formula:

(1) $$N=\frac{Z^{2}P\left(1-P\right)}{e^{2}}$$

Where N = sample size, Z = level of confidence, P = baseline level of the selected indicator and e = margin of error, set at P = 0.50, Z = 1.96 (at 95% confidence interval), e = 0.05.

The sample size was adjusted for 1.5 (multiply) design effect for multiple sites, 20% (divide by 0.8) refusal rate and 4 (multiply) ten-year interval for 5–14, 15 years or more age groups for both males and females.

The minimum required sample size after adjusting for the above factors was therefore 2,610 people to be enrolled to determine the population-based prevalence of leprosy. Each district was therefore to recruit at least 652 people and each site (EA) in the district at least 130 people.

#### Eligible participants and recruitment process

All people aged 5 years or more in the selected EAs were eligible to participate in the study. Chiefs and community health workers known as Health Surveillance Assistants (HSAs) from the selected EAs were oriented on leprosy and on their role to raise awareness. On the day of the study, the chiefs and HSAs mobilised the people at one place. The recruitment process started with health talk on common skin conditions and leprosy. People were then informed about the purpose of the study and then asked to participate voluntarily. Written informed consent was obtained from those that were willing to take part in the study and be examined physically. Consenting clients were enrolled using a standard enrolment form. The enrolment form contained social and demographic information (name, age, sex, education etc.). After registration, clients went for medical history taking and physical examination conducted by trained and experienced leprosy and skin clinicians. Physical examination was conducted in shelters to ensure privacy. Clients with skin diseases were given medical treatment.

### Diagnosis of leprosy

Leprosy was diagnosed based on the presence of leprosy cardinal signs. A person was diagnosed as leprosy case if he/she had one or more hypo-pigmented (whitish or brownish) skin patches with loss of sensation in the patch and/or enlargement of peripheral nerves and/or was currently on leprosy treatment with multidrug therapy. Leprosy patches could be pale or reddish, could be flat or raised, do not itch, usually painless, lack sensation to touch, pain or heat and could appear anywhere on the body. Other signs of leprosy include reddish or skin-coloured nodules or smooth, shiny diffuse thickening of the skin without a loss of sensation [5]. The identified new cases of leprosy were registered with the district leprosy programme and started on treatment. Leprosy cases currently on treatment were assessed for compliance to treatment. Those that were not compliant were counseled and re-started on treatment. Those that were compliant were encouraged and thanked.

### Part two of the study: Evaluation of Malawi National Leprosy Elimination Programme

World Health Organisation guidelines and tools for leprosy elimination monitoring (LEM) were used to evaluate the Malawi National Leprosy Elimination Programme [11]. National and district leprosy data and drug stocks (MDT blister packs, prednisolone and loose clofazimin tablets) in the selected districts were reviewed. In each district, district hospital and three health centres were visited where leprosy data, patients on treatment and/or people on prevention of disabilities care were reviewed. Health workers (medical assistants) were interviewed to assess their capacity in diagnosis and treatment of leprosy. WHO LEM forms and questionnaires were used to collect data at national and district level. The whole study (part one and part two) was conducted from October to December 2011.

### Data management

Data from leprosy active surveillance and promotion of community awareness were entered in Epi Info 2004 version 3.2.2 (Centre for Disease Control, Atlanta Ga) and exported to SPPS for windows version 11.0.0 (Chicago, IL) for analysis. Confidence intervals (CI) for proportion were calculated using the formula p = p ± C√p(p-1)/n where p is the given proportion whose CI needs to be calculated, C = is the coefficient, at 95%CI C = 1.96, n = number of participants. The results were statistically significant if there was no overlap between two CIs of comparing groups.

Data from evaluation of national leprosy elimination programme were entered and analysed using WHO LEM tools. Data were analysed to build the three groups of LEM indicators as follows:

#### Elimination indicators

Prevalence, detection, prevalence/detection ratio, trends of detection, multibacillary (MB), children and grade 2 disabilities in new cases and other admitted cases (relapses, retrieved defaulters, transferred in).

#### Integration indicators

Percentages of health facilities providing MDT, accessibility of MDT (distance, costs, availability of blister packs, flexibility of supervision of MDT monthly intakes).

#### Quality indicators of MDT and other leprosy services

Quality and quantity of blister packs, cure rate of MB cohort of 2009 and paucibacillary (PB) cohort of 2010 and other indicators (prevention of disabilities activities and availability of prednisolone and loose clofazimin tablets).

## Results

### Characteristics of participants enrolled in leprosy active surveillance and promotion of community awareness

A total of 6,338 people participated in part one of the study of whom 60.2% were females and 35.2% were children aged less than 15 years old. Table 1 shows characteristics of participants enrolled in leprosy active surveillance and promotion of community awareness.

**Table 1:** Characteristics of participants enrolled in leprosy active surveillance and promotion of community awareness in four districts in Malawi

	**Male**		**Female**		**Total**	
	**n**	**%**	**n**	**%**	**n**	**%**
**Sex**	2524	39.8	3814	60.2	6338	100.0
**Age (years):**						
5-14	1106	44.1	1111	29.3	2217	35.2
15-24	397	15.8	888	23.5	1285	20.4
25-34	326	13.0	791	20.9	1117	17.7
35-44	204	8.1	361	9.5	565	9.0
45-54	153	6.1	281	7.4	434	6.9
55-64	139	5.5	204	5.4	343	5.5
65-74	116	4.6	107	2.8	223	3.5
75 or more	66	2.6	43	1.1	109	1.7
All with known age	2507	100.0	3786	100.0	6293	100.0
**Marital status:**						
Currently married	980	74.1	1597	69.3	2577	71.0
Divorced/separated	59	4.5	185	8.0	244	6.7
Never married	264	20.0	350	15.2	614	16.9
Widowed	19	1.4	174	7.5	193	5.3
All with known marital status	1322	100.0	2306	100.0	3628	100.0
**District:**						
Machinga	738	29.2	1478	38.8	2216	35.0
Mangochi	421	16.7	764	20.0	1185	18.7
Nkhotakota	540	21.4	646	16.9	1186	18.7
Salima	825	32.7	926	24.3	1751	27.6
All	2524	100	3814	100	6338	100

Key: n = number of participants in the group, % = percentage.

### Population-based prevalence of skin diseases

Overall, 18% of 6338 people examined had skin diseases. The commonest skin disease was fungal (9%) followed by eczema/dermatitis (3%) and leprosy (1%). Of the fungal skin conditions, pityriasis versicolor and Tinea capatis were the commonest (22% and 21% respectively) followed by Tinea corporis (9%), Tinea cruris (6%) and Tinea pedis (2%). Skin diseases (all types) and fungal skin diseases were more frequent in males than females (20% vs 16%, 10% vs 8% respectively, all *p* < 0.05), bacterial skin infections in children aged 5–14 years old than adults aged 15 years or more (1.7% vs 0.5%, *p* < 0.05), leprosy in adults than children (1.3% vs 0.4%, *p* < 0.05) (table 2).

**Table 2:** Community camp-based prevalence of and skin diseases in four districts in Malawi, 2011

	**All (n = 6338)**	**Male (n = 2524)**		**Female (n = 3814)**		**Age 5–14 years (n = 2217)**		**Age 15 years or more (n = 4076)**	
	**%**	**%**	**95%CI**	**%**	**95%CI**	**%**	**95%CI**	**%**	**95%CI**
Fungal skin diseases	8.7	10.2*	9.0-11.4	7.7	6.9-8.5	7.5	6.4-8.6	9.3	8.4-10.2
Eczema/dermatitis	2.9	3.5	2.8-4.2	2.4	1.9-2.9	3.5	2.7-4.3	2.5	2.0-3.0
Leprosy	1.0	1.4	0.9-1.9	0.8	0.5-1.1	0.4	0.1-0.7	1.3*	1.0-1.7
Bacterial skin infections	0.9	1.2	0.8-1.6	0.7	0.4-1.0	1.7*	1.2-2.2	0.5	0.3-0.7
Viral skin infections	0.2	0.2	0.03-0.4	0.2	0.06-0.3	0.3	0.07-0.5	0.2	0.06-0.3
Parasitic skin infection	0.2	0.3	0.09-0.6	0.1	0.0-0.2	0.1	0.0-0.2	0.2	0.1-0.3
Other skin diseases	3.8	3.5	2.8-4.2	3.9	3.3-4.5	2.9	2.2-3.6	4.2	3.6-4.8
All	17.7	20.4*	18.8-22.0	15.9	14.7-17.1	16.5	15.0-18.1	18.2	17.0-19.4

Key: n = number of participants in the group, % = percentage, CI = confidence interval, * statistically significant, p <0.05; male vs female, age 5-14 years vs age 15 years or more.

### Population-based prevalence of leprosy

A total of 66 leprosy cases were identified out of 6338 people that were examined, giving a prevalence of 104.1 per 10,000 population. Mangochi district had the highest prevalence (194.1) followed by Salima (97.1), Nkhotakota (92.1) and Machinga (67.1). Of the 66 leprosy cases, 37 were new cases, 6 were defaulters and 23 were currently on treatment, 30 were females and 9 were children aged less than 15 years old. Of the 37 new cases, 9 (24.3%) were children, 25 (68.4%) had 1–5 leprosy lesions (paucibacillary) and 8 (21.1%) had grade 2 disability. The most frequent location of leprosy lesion on the body was the head and neck (24.1%), arms (24.1%), chest (17.2%), legs (13.8%), back (13.8%) and abdomen (7.0%). Table 3 shows characteristics of leprosy cases identified through active surveillance and promotion of community awareness.

**Table 3:** Community camp-based prevalence of leprosy in four districts in Malawi, 2011

**District**	**No. of people screened**	**No. of new leprosy cases identified**	**No. of leprosy cases who defaulted treatment identified**	**No. of leprosy cases currently on treatment**	**Total number of leprosy cases**	**Estimated leprosy prevalence per 10,000 population (in study population)**
Machinga	2216	6	4	5	15	67.1
Mangochi	1185	12	0	11	23	194.1
Nkhotakota	1186	9	0	2	11	92.1
Salima	1751	10	2	5	17	97.1
All	6338	37	6	23	66	104.1

### Trends of leprosy indicators at national level and in selected districts: 2006–2011

Leprosy data were reviewed from 4 district hospitals and 12 health centres. In addition, 17 people affected by leprosy, 2 district leprosy coordinators and 9 medical assistants were interviewed to assess knowledge on leprosy diagnosis, treatment and prevention of disabilities.

**Figure 1:**
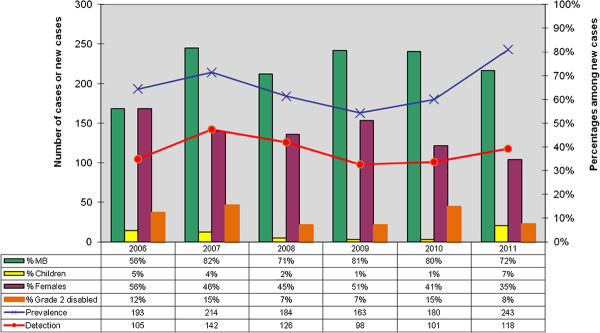
Trends of leprosy key indicators in four selected districts in Malawi 2006-2011.

**Figure 2:**
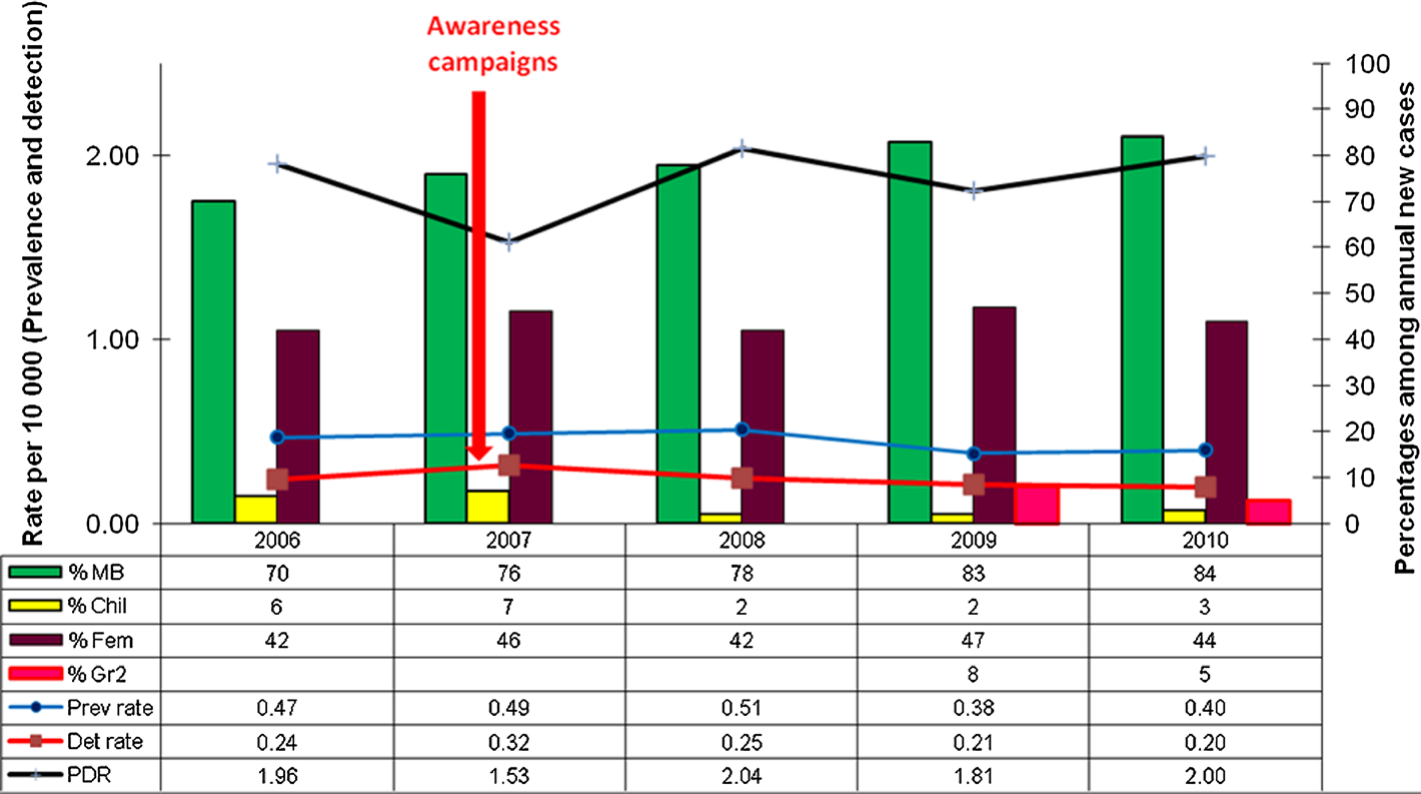
Trends of Leprosy key indicators in MALAWI 2006-2010.

In the selected districts, from 2006 to 2011, trends of prevalence and detection increased in 2007 (because of awareness campaigns which were conducted) then decreased until the year 2009. In 2010 and 2011, the two trends increased to a total number of 243 cases for the prevalence and 118 new cases for the detection in 2011. The increase was observed mainly in Nkhotakota district (116 new cases), Mangochi (57 new cases) and Salima (45 new cases). At the end of November 2011, prevalence was higher than the leprosy elimination threshold in Nkhotakota and Salima at 3.54 and 1.2 leprosy cases per 10,000 population respectively. However, at National level leprosy prevalence remained less than 1 case per 10,000 inhabitants. Percentages of MB, females, children and grade 2 disabilities among new cases showed no clear increasing or decreasing trends and could not be interpreted in terms of early diagnosis, reduced transmission of the disease in communities and progress towards elimination of leprosy (Figure 1 and Figure 2).

Integration of leprosy elimination activities at health centre level was limited in all the 12 health centres visited. In general, medical assistants in charge of the leprosy programme at health centre level were not formally trained on leprosy elimination activities. Diagnosis, treatment and patient drug collection were only done at the district hospitals by leprosy district coordinators or dermatology officers.

Availability of MDT blister packs in the visited health facilities were sufficient (3 months for MB adult, 59 months for MB child, 11 months for PB adults and 10 months for PB child). There was a risk of expiry mainly for child blister packs for both MB and PB. Average cure rates for 2009 MB and 2010 PB cases, for the 4 districts were 33% and 63% respectively.

## Discussion

In 1991 the World Health Assembly decided to eliminate leprosy as a public health problem by the year 2000. Elimination was defined as reducing the global prevalence of the disease to less than 1 case per 10,000 population. In 2000 the World Health Organization (WHO) announced that leprosy was eliminated globally [12]. However, leprosy still remains a significant public health problem at national and sub-national levels in some endemic countries [13]. The Malawi Leprosy Elimination Programme achieved the WHO defined leprosy elimination as a public health problem at the end of 1994. After this achievement, the country did not benefit from special support from WHO Leprosy Elimination Programme, which concentrated its support on the “final push strategy” to countries that had not achieved the leprosy elimination goal. This led to the reduction of support received from partners and to the progressive withdrawal of financial and technical support of LEPRA-UK from 1996 to 2009. Since then, the programme performed sub- optimally in essential components such as case finding, treatment with WHO-recommended MDT, prevention of disabilities and monitoring the programme performance through a sound recording, registration and reporting system.

Findings from this study confirmed the reduced performance of the programme and that high numbers of leprosy cases were occurring in some districts. Population-based prevalence estimates in all the four selected districts were off WHO elimination target. The estimated prevalence per 10,000 population in the study population ranged from 67 in Machinga to 194 in Mangochi. These estimates were higher than previously reported in Karonga (40–60) in 1980s [14,15]. Among 37 new cases identified at community level, 9 were children aged less than 15 years old suggesting that the disease was being transmitted in the communities where the children came from [16,17]. Prevalence/detection ratios were over 1 for all the years between 2006 and 2011, indicating poor case-holding of leprosy patients, poor recording and updating of leprosy registers at district level. Integration of leprosy activities in health centres was partial, contributing to long delay in diagnosis and longer periods of MDT treatments. Consequently, cure rates by cohort analysis of 2009 MB and 2010 PB cases were 33% and 63% respectively, far below the expected 80%. High leprosy MB ratio in the passive health facility-based data has been shown that it is not a sign of positive impact as previously thought but of severe under reporting to the extent of 73% when MB ratio reaches 47.5% [18]. In Malawi, it could be even more since the proportion of MB cases in passive data was 88%, higher than in India which was at about 50%.

This study also demonstrated that active surveillance promotes early detection, treatment and gender balance in accessing leprosy services thereby reducing the risk of disability and stigma. This observation was consistent with other findings of modified leprosy elimination campaigns [19,20]. Traditional house-to-house leprosy elimination campaign is expensive and unsustainable in resource- poor settings and is no longer encouraged by WHO [17]. However, it could be modified to reduce the cost and maximise the health benefits [19]. In this study people were mobilised to one place rather than health workers moving from house-to-house and were examined and treated for all skin diseases not just leprosy thereby maximizing the health benefits. Based on the facility-based data, there was no child case of leprosy from this population. The active community-based surveillance detected and put on treatment 9 children with leprosy. This suggested that child cases of leprosy were particularly missed by the facility-based self-reporting of cases. Information provided by this study on the distribution of leprosy lesions on the body is useful to health workers. Up to 62% of leprosy lesions were on the face, arms, legs where they could be easily seen in any person attending health facility irrespective of the reason for visiting the health facility. Enabling health workers to recognise leprosy lesions could make them to utilise the high attendance rate in public hospitals as an opportunity to detect leprosy cases that may have gone the health facility for a different reason.

Recent population-based prevalence estimates of skin conditions are not well documented in Malawi except 1980s data from Karonga [21,22]. This study therefore provided an update that skin conditions were still a major public health problem affecting 18% of the population aged 5 years or more. Our findings that fungal infections, pityriasis versicolor in particular being the commonest skin infections were consistent with previous findings [21]. Previous trials indicated that Whitfield's and clotrimazole creams were effective for the treatment of common fungal skin infections in Malawi with cure rates of 80%-90%. The lower cost made Whitfield's cream the treatment of choice for fungal infections of the skin in primary health care [23]. Both Whitfield's and clotrimazole creams still seemed to be effective although the cure rates were currently unknown.

In summary, this study revealed the following challenges facing the Malawi Leprosy Elimination Programme: increasing and high prevalence which were over the leprosy elimination goal, high prevalence/detection ratios, lack of decentralisation to health centres for diagnosis and treatment, insufficient knowledge and skills of health centre staff and poor registration and recording system. There was therefore need for renewed political will and commitment by both government and partners for resources for the final push to eliminate leprosy at national and district level. The final push involves improving community awareness on signs and symptoms and dispelling the fear of leprosy, motivating people to seek treatment, enabling health workers to diagnose and treat patients early and ensuring that all patients are cured using multidrug therapy [5,17].

### Limitations of the study

The second step of the sampling process where people were mobilised at one place in the community could be the source of bias because it depended on the compliance. People who thought had leprosy might have selectively came forward thereby leading to over-estimation or might have stayed away for fear of being stigmatised thereby leading to under-estimation of leprosy prevalence. Chiefs and community health workers were involved in the mobilisation and promotion of community awareness for sustainability of leprosy elimination efforts. While recognizing the shortfall of mobilising people to one place rather than random selection of households and eligible participants, the method was more appropriate for promotion of community awareness, involvement and openness on leprosy. More people (more than twice the sample size) participated making it more representative of the study population. Over-representation of females (60% of participants were females) was another limitation of this study. Relatively fewer males participated in the study because there were away working in the fields as the study population was predominantly subsistence farmers. It was not known whether this group had different study characteristics.

## Conclusion

Leprosy was still an important public health problem and was getting out of control in some districts in Malawi. Renewed commitments and efforts were needed to sustain leprosy elimination at national and district level. Improving knowledge and skills of health workers particularly those working in health centres, registration and recording of data, contact tracing, decentralisation and integration of treatment to health centres and introduction of leprosy awareness days and community-based surveillance could help to improve early detection, treatment, case holding and prevention of disabilities and stigma.

## Competing of interests

The authors declare that they have no competing interests.

## Author Contributions

Conceived and designed the study: KPM, LRM, HWK. Performed the study: KPM, LRM, HWK, DN, ML, SM, WWS, PM. Analyzed the data: KPM, LRM, HWK. Contributed reagents/materials/analysis tools: KPM, HWK. Wrote the paper: KPM, LRM, HWK, DN, ML, SM, PPM, WWS. "All authors read and approved the final manuscript.

## Pre-publication history

The pre-publication history for this paper can be accessed here:

http://www.biomedcentral.com/1472-698X/12/12/prepub
